# A23 INVESTIGATING THE LINK OF BACTERIAL TRANSLOCATION AND BEHAVIORAL CHANGES IN A MOUSE RESTRAINT STRESS MODEL

**DOI:** 10.1093/jcag/gwae059.023

**Published:** 2025-02-10

**Authors:** C Shimbori, A Khalil, N Kraimi, J Lu, G De Palma, S Hapfelmeier, S Collins, P Bercik

**Affiliations:** McMaster University, Hamilton, ON, Canada; McMaster University, Hamilton, ON, Canada; McMaster University, Hamilton, ON, Canada; McMaster University, Hamilton, ON, Canada; McMaster University, Hamilton, ON, Canada; Universitat Bern Institut fur Infektionskrankheiten, Bern, Bern, Switzerland; McMaster University, Hamilton, ON, Canada; McMaster University, Hamilton, ON, Canada

## Abstract

**Background:**

Bacterial translocation is defined as migration of bacteria or bacterial fragments from the intestinal lumen into extraintestinal tissues; this process has been implicated in the pathophysiology of gastrointestinal disorders and central nervous system disorders. However, the direct causal relationship between bacterial translocation and behavioral changes, as well as the mechanism of bacterial translocation remains unclear. In the previous study, we showed that commensal microbes can cross the intestinal barrier and access deeper layers of the gut and distal organs in the restraint stress model. In this study, we further investigated whether bacterial translocation contribute to behavioral changes in a mouse restraint stress model using invasive bacteria.

**Aims:**

To investigate whether invasive bacteria can translocate and exacerbate behavioral alterations in a stress model.

**Methods:**

SPF C57BL/6 mice were assigned to either the restraint stress group or the non-stress control group. Each group was further divided into two subgroups: one receiving a vehicle and the other inoculated with auxotrophic Salmonella enterica serovar Typhimurium HA630 via oral gavage three times per week during the stress period (n=6-14 per group). In a separate experiment, stressed and non-stressed mice were each split into 2 groups: vehicle or GFP-Salmonella; inoculated with GFP labeled wild type *Salmonella enterica* serovar *typhimurium* via single oral gavaging (n=6-7). After the last session of stress, we conducted light/dark preference and tail suspension tests. We collected jejunum, colon, myenteric lymph node, liver, spleen, and brain samples for histology and performed iterative bleaching extends multiplexity (IBEX) staining. We also sampled blood, liver, spleen, and brain for bacterial cultures under aseptic conditions.

**Results:**

Transient salmonella inoculation enhanced stress-induced anxiety-like behaviour accompanied by higher rate of bacterial translocation. Histology analysis showed that intestinal bacteria were invade into the intestinal mucosa and co-localized with goblet cells in jejunum, and colon, and some of them were in close proximity with neurons in stress group. Interestingly, some bacteria appeared to be within the CD103^+^ cells in the colon, as well as in the hippocampus. GFP-salmonella inoculation enhanced stress-induced depression-like behaviour accompanied by higher rate of bacterial translocation.

**Conclusions:**

This study indicates that intestinal bacteria translocation could contribute to the stress-induced behaviors. Our histology data suggest potential contribution of goblet and CD103^+^ cells in gut bacterial translocation in the restrained stress model.

**Funding Agencies:**

CAG, CIHR

